# Exploratory open-label clinical study to determine the S-588410 cancer peptide vaccine-induced tumor-infiltrating lymphocytes and changes in the tumor microenvironment in esophageal cancer patients

**DOI:** 10.1007/s00262-020-02619-3

**Published:** 2020-06-04

**Authors:** H. Daiko, T. Marafioti, T. Fujiwara, Y. Shirakawa, T. Nakatsura, K. Kato, I. Puccio, T. Hikichi, S. Yoshimura, T. Nakagawa, M. Furukawa, K. Stoeber, M. Nagira, N. Ide, T. Kojima

**Affiliations:** 1 Esophageal Surgery Division, National Cancer Center Hospital, 5-1-1, Tsukiji, Chuo-ku, Tokyo, 104-0045 Japan; 2 Department of Cellular Pathology, University College London Hospital, London, UK; 3 Department of Gastroenterological Surgery, Okayama University Graduate School of Medicine, Dentistry and Pharmaceutical Sciences, Okayama, Japan; 4 Division of Cancer Immunotherapy, Exploratory Oncology Research and Clinical Trial Center, National Cancer Center, Kashiwa, Japan; 5 Gastrointestinal Medical Oncology Division, National Cancer Center Hospital, Tokyo, Japan; 6 R&D Department, Cancer Precision Medicine, Inc., Kawasaki, Japan; 7 Drug Discovery and Disease Research Laboratory, Shionogi & Co., Ltd., Toyonaka, Japan; 8 Biostatistics Department, Shionogi & Co., Ltd., Osaka, Japan; 9 Business Development, Shionogi & Co., Ltd., London, UK; 10 Project Management Department, Shionogi & Co., Ltd., Osaka, Japan; 11 Department of Gastroenterology and Gastrointestinal Oncology, National Cancer Center Hospital East, Kashiwa, Japan

**Keywords:** Tumor-infiltrating lymphocytes, PD-1, PD-L1, Cancer peptide vaccine, Esophageal cancer

## Abstract

Cancer vaccines induce cancer-specific T-cells capable of eradicating cancer cells. The impact of cancer peptide vaccines (CPV) on the tumor microenvironment (TME) remains unclear. S-588410 is a CPV comprising five human leukocyte antigen (HLA)-A*24:02-restricted peptides derived from five cancer testis antigens, DEPDC1, MPHOSPH1, URLC10, CDCA1 and KOC1, which are overexpressed in esophageal cancer. This exploratory study investigated the immunologic mechanism of action of subcutaneous S-588410 emulsified with MONTANIDE ISA51VG adjuvant (median: 5 doses) by analyzing the expression of immune-related molecules, cytotoxic T-lymphocyte (CTL) response and T-lymphocytes bearing peptide-specific T-cell receptor (TCR) sequencing in tumor tissue or blood samples from 15 participants with HLA-A*24:02-positive esophageal cancer. Densities of CD8+, CD8+ Granzyme B+, CD8+ programmed death-1-positive (PD-1+) and programmed death-ligand 1-positive (PD-L1+) cells were higher in post- versus pre-vaccination tumor tissue. CTL response was induced in all patients for at least one of five peptides. The same sequences of peptide-specific TCRs were identified in post-vaccination T-lymphocytes derived from both tumor tissue and blood, suggesting that functional peptide-specific CTLs infiltrate tumor tissue after vaccination. Twelve (80%) participants had treatment-related adverse events (AEs). Injection site reaction was the most frequently reported AE (grade 1, *n* = 1; grade 2, *n* = 11). In conclusion, S-588410 induces a tumor immune response in esophageal cancer. Induction of CD8+ PD-1+ tumor-infiltrating lymphocytes and PD-L1 expression in the TME by vaccination suggests S-588410 in combination with anti-PD-(L)1 antibodies may offer a clinically useful therapy.

*Trial registration* UMIN-CTR registration identifier: UMIN000023324.

**Electronic supplementary material:**

The online version of this article (10.1007/s00262-020-02619-3) contains supplementary material, which is available to authorized users.

## Introduction

Esophageal cancer is the seventh most common cancer, with a global incidence of around 570,000 and 510,000 deaths annually—accounting for 1 in 20 cancer deaths—with the highest age-standardized incidence rate among East Asian men [1]. Data from the National Cancer Center in Japan described 22,710 new esophageal cancer cases and 11,576 deaths in 2014 [2].

Esophageal cancer has limited effective treatment options. Current clinical practice guidelines recommend chemotherapy with 5-fluorouracil, platinum and a taxane, with recent advances in cancer immunotherapies offering promising options [3]. Administration of the antiprogrammed death-1 (PD-1) antibodies nivolumab and pembrolizumab reduced tumor burden in advanced esophageal cancer in phase 2 studies, but survival benefit was limited [4, 5]. However, a limited (2.5 months), but statistically significant survival benefit was observed with nivolumab versus investigator’s choice of chemotherapy in the phase 3 ATTRACTION-3 study of patients with unresectable advanced or recurrent esophageal squamous cell carcinoma that was refractory or intolerant to one previous fluoropyrimidine-based and platinum-based chemotherapy, which led to the approval of nivolumab in this indication [6]. Treatment options have increased, but there is still an urgent need to explore other therapeutic approaches to improve survival in esophageal cancer patients.

Cancer peptide vaccines (CPVs) aim to enhance the host immune response to cancer antigens. Multiple peptides included in CPVs have been shown to induce both cytotoxic T-lymphocytes (CTLs) in blood and a clinical response in patients with esophageal squamous cell carcinoma [7, 8]. These studies also suggest an overall survival benefit may be seen in patients with CTL responses to a higher number of peptides in CPVs [7, 8]. However, it is difficult to elucidate the relationship between antitumor efficacy and the mechanism of action of CPVs with blood-based immune monitoring alone.

Tumor-infiltrating lymphocytes (TILs) are implicated in cancer immunotherapy, with the efficacy of antiprogrammed death-ligand 1 (PD-L1) antibodies being dependent on the presence of pre-existing TILs. For example, the presence of TILs and immune-related molecules, such as PD-L1, in the tumor microenvironment (TME) among patients with esophageal cancer was associated with better survival [9]. Recently, the existence of similar sequences of T-cell receptors (TCRs) on CTLs and TILs was recorded in patients injected with glypican-3 peptide [10]. These findings imply that CPVs have the potential to induce not only CTLs, but also TILs, in esophageal cancer.

S-588410 is a novel CPV comprising five human leukocyte antigens (HLA)-A*24:02-restricted 9–10-mer peptides derived from five cancer testis antigens (DEPDC1, MPHOSPH1, URLC10, CDCA1 and KOC1), all of which have been found to be upregulated in esophageal cancer [11, 12]. CPVs comprising any one of these five peptides have been reported to generate peptide-specific CTLs in several cancer types, including esophageal cancer [7, 8, 11–14]. As it is possible to biopsy esophageal cancer, a neoadjuvant trial design is also feasible where CPV is administered before surgical resection.

This exploratory study aims to determine the tumor immune response of S-588410 in the TME. The primary objective was to evaluate the effects of S-588410 administration on the CD8+ TIL density in esophageal cancer tissue from participants with planned surgical resection. Secondary objectives were to evaluate the safety of S-588410 administration and CTL induction potency in peripheral blood samples.

## Materials and methods

### Study design

**Fig. 1:**
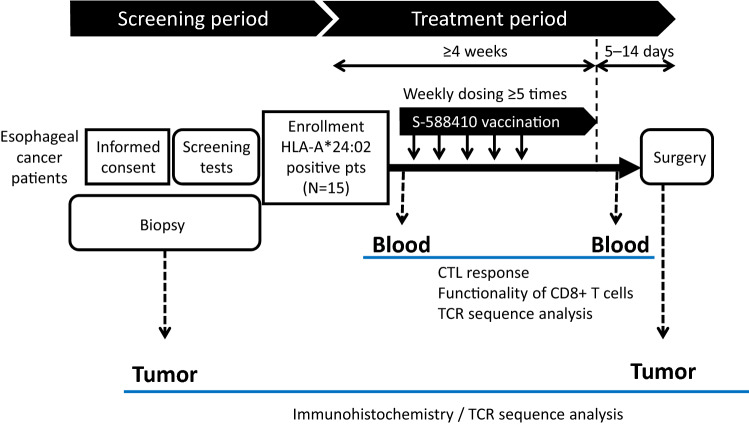
Study design

This was a prospective, open-label, single-arm exploratory study conducted in three medical centers in Japan. Figure 1 shows a schematic of the study design. S-588410 was emulsified with the adjuvant MONTANIDE ISA51VG (Seppic S.A., Paris, France) for a final concentration of 1 mg/1 mL for each peptide.

A 1-mL dose of the emulsion was injected subcutaneously once weekly, with each participant receiving at least five doses, with a minimum of 5 days between last dose and esophagectomy. Blood and tumor tissue samples were obtained before and after S-588410 administration. The primary endpoint was percent change in CD8+ TIL density in tumor tissue samples. Secondary endpoints included the safety of S-588410 administration and the presence or absence of CTL induction in blood samples. Further exploratory endpoints included the expression of immune-related molecules in tumor tissue and peptide-specific TCR repertoire.

This study was approved after review by the relevant regulatory and independent ethics committees at each study site and was conducted in accordance with the Declaration of Helsinki and International Conference on Harmonization Good Clinical Practice. All participants provided written informed consent before enrollment. This study was prospectively registered on the University Hospital Medical Information Network Clinical Trials Registry (UMIN-CTR) prior to enrollment of the first participant on August 1, 2016 (Study identifier: UMIN000023324).

### Participants

Eligible participants were aged ≥ 20 years, had HLA-A*24:02-positive blood tests and histologically confirmed esophageal squamous cell carcinoma or adenocarcinoma who were scheduled for esophagectomy at least 30 days after first administration of study drug. Pregnant or lactating women, and those scheduled to receive, or having previously received, anticancer drugs, radiotherapy, thermotherapy, systemic immunosuppressants or immunotherapy for primary esophageal cancer were excluded from the study. Other exclusion criteria were serious concurrent disease (such as a hepatic disorder, a renal disorder, cardiac disease, hematologic disease, respiratory disease or metabolic disease) or if laboratory tests indicated impaired bone marrow, hepatic or renal function within 28 days of enrollment. Participants with a history of serious allergic reactions related to administration of drugs, vaccines or biologics, or with autoimmune disease, immunodeficiency disease, uncontrollable systemic or active infection were also excluded. Participants who had previously received any of the five peptides included in S-588410 or other investigational products within 28 days (or 5-times the product’s half-life) prior to enrollment were also excluded.

### Safety assessment

Investigators assessed the injection site, vital signs, electrocardiogram and clinical laboratory blood tests of participants at defined time points (Fig. 1). New-onset or aggravated adverse events (AEs) were assessed and graded by investigators according to Common Terminology Criteria for Adverse Events (CTCAE) Version 4.03. AEs of special interest included injection site reactions, eosinophilic pneumonia, influenza-like symptoms and anaphylactic reactions.

### Tissue preparation for immune-related molecule expression analysis

Tissue samples were taken via core needle biopsy (pre-vaccination) or by surgery (following vaccination), with samples embedded in paraffin and microsectioned onto slides for immunohistochemistry (IHC) with three anti-immune-related molecule antibodies in four panels (Supplemental Methods 1). IHC-prepared tissue samples underwent digital image analysis using DEFINIENS (Munich, Germany) proprietary software [15]. IHC analysis determined CD8+ count, as well as confirming expression of CD4, CD25, FoxP3, Granzyme B, PD-1 and PD-L1. Percent change in CD8+ cell density was calculated using the equation: ((CD8+ cell density in the tissue sample at surgery minus CD8+ cell density in the tissue sample at biopsy)/CD8+ cell density in tissue sample at biopsy) × 100. Expression of HLA class I and the five antigen proteins was also evaluated in tumor tissue post-vaccination. The list of antibodies and scoring methods is described in Supplemental Methods 2.

### Blood sample preparation for CTL analysis

Peripheral blood mononuclear cells (PBMCs) were obtained from blood samples taken before and after S-588410 administration. CTL activity specific for each of the five peptides in S-588410 was determined by assessing spot count of interferon γ (IFN-γ)-generating cells using an enzyme-linked immunospot (ELISPOT) assay after in vitro stimulation (IVS) of PBMCs with cognate peptides for 1 week [13]. The number of spots was classified into four grades (−, +, ++, +++), and the peptide-specific CTL response rate was calculated using the equation: number of participants who graded up after S-588410 administration/total number of participants.

### Flow cytometry

CD8+ T-cells in cryopreserved PBMCs were assessed using flow cytometry to detect molecular markers for CD3 and CD8 positivity and CD14, CD19 and CD54 negativity. Peptide-specific functionality was assessed following staining by anti-PD1 antibody and URLC10-peptide tetramer or DEPDC1-peptide tetramer. All stained cells were analyzed on a FACS Canto flow cytometer (BD Biosciences, San Jose, CA, USA). Data analysis was performed using FlowJo version 10.5 (TreeStar, San Carlos, CA, USA). Detailed methodology and stains used are described in Supplemental Methods 3.

### TCR repertoire analysis

Total ribonucleic acid (RNA) was extracted from tumor tissue and PBMCs pre- and post-vaccination, as well as sorted fraction of tetramer + CD8+ T-cells after IVS culture using RNeasy mini kit or RNeasy micro kit (QIAGEN, Hilden, Germany). Peptide-specific TCR sequences were identified in the fraction of tetramer + CD8+ T-cells, and their frequency was tracked in tumor tissue and PBMCs pre- and post-vaccination. TCR tracking was performed using software (ImmunoGrapher) developed by Cancer Precision Medicine, Inc. (Kawasaki, Kanagawa, Japan). A schematic for the TCR analysis and detailed methods is described in Supplemental Figure 1 and Supplemental Methods 4.

### Statistical analysis

Statistical analysis was conducted in the full analysis set, comprising all enrolled participants who received study drug and had tissue or blood samples available before and after vaccination. In addition, a post hoc analysis comparing the density of immune-related molecule-expressing cells before and after administration used a Wilcoxon signed-rank test with a two-tailed significance level of 0.05. Statistical analysis was conducted using SAS version 9.2. A target sample size of 15 participants was selected based on ethical considerations and feasibility of recruitment, given that no statistical estimation requirements were necessary for the primary and secondary endpoints.

## Results

### Participants and safety assessment

Fifteen HLA-A*24:02+ participants with esophageal squamous cell carcinoma scheduled for surgical resection were enrolled between September 1, 2016, and December 6, 2017. Participant characteristics are summarized in Table 1.

**Table 1:** Patient characteristics and clinical response to S-588410 administration

Patient no.	Age (years)	Sex	Type of HLA-A	Stage^a^	Tumor location	S-588410 doses administered, *n*	Vaccine peptides inducing CTL, *n*
1CB003	76	M	0201/2402	IB	Mid	5	1
1CB005	68	M	2402/3101	IA	Mid	3	1
1CB011	78	M	2402/3101	IA	Mid	6	2
1CB012	71	M	2402/3303	IA	Mid	5	2
1CB013	76	M	2402/3101	IIB	Mid	6	1
1CB014	79	M	2402/2402	IA	Mid	5	2
1CB020	69	M	2402/3101	IA	Mid	8	3
1CB023	73	F	2402/2402	IA	Mid	4	2
1CB025	73	M	0206/2402	IA	Mid	6	2
1FB003	65	M	0201/2402	IA	Mid	5	3
1FB006	57	M	2402/3303	IA	Mid	14	1
1FB010	79	M	2402/3101	IA	Lower	11	2
1FB011	66	M	2402/3101	IA	Upper	5	2
1FB012	64	M	0206/2402	IA	Lower	8	1
1FB013	62	M	2402/2402	IA	Mid	5	2

*CTL* cytotoxic T-lymphocyte, *HLA* human leukocyte antigen, *UICC* Union for International Cancer Control

^a^Stage according to the 7th Edition of the Union for International Cancer Control TNM Classification of Malignant Tumors for esophageal cancer

The majority of participants (14/15, 93%) were male, with a median age of 71 (range 57–79) years. Most cancers were stage IA, according to the 7th Edition of the Union for International Cancer Control TNM Classification of Malignant Tumors, occurring in the mid-thoracic esophagus. Participants received between three and 14 injections of S-588410, with a median of five injections. In total, 87% (13/15) of participants received at least the five planned doses.

Treatment-related AEs occurred in 80% of participants (12/15). No anaphylactic reactions or deaths were recorded during the study. Influenza-like symptoms were rare, with nasopharyngitis and sputum retention reported by one participant (6.7%). Grade 1 pruritus was reported by one participant (6.7%). Injection site reactions were reported by 12 participants (80%), of which one event was classified as Grade 1 and the remaining 11 classified as Grade 2. One participant (1CB005) discontinued due to an injection site reaction. This participant experienced a Grade 2 injection site reaction characterized by redness and induration without pruritus or ulceration. One further participant had study drug withdrawn due to worsening gall bladder cancer classified as unrelated to study drug by the investigator. Another participant withdrew from the study due to an accelerated surgery schedule.

### Immune response analysis in tumor tissue

**Fig. 2:**
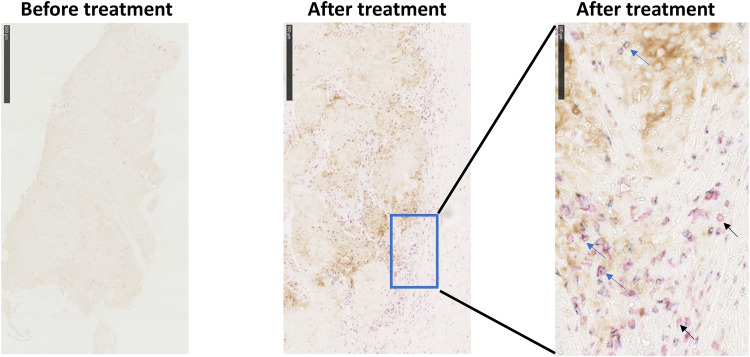
Immunohistochemical staining in representative samples of tumor tissue from a patient with esophageal cancer before and after S-588410 vaccination. CD8, Granzyme B and programmed death-ligand 1 (PD-L1) are stained red, blue and brown, respectively. Black arrows identify CD8+ cells. Blue arrows identify CD8+Granzyme B+ cells

**Fig. 3:**
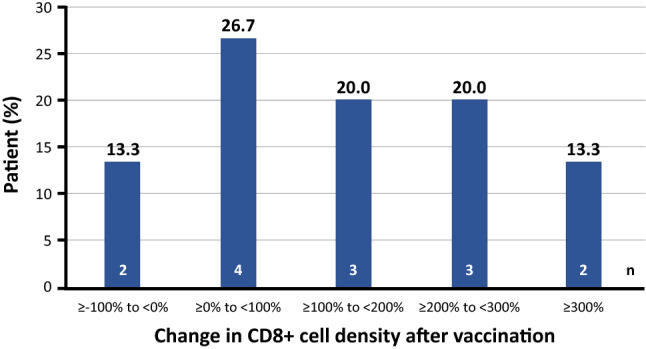
Percent change in CD8+ cells densities in tumors after S-588410 vaccination. Change in the level of CD8+ cell density was calculated with the equation: ((CD8+ cell density in the tissue sample at surgery minus CD8+ cell density in the tissue sample at biopsy)/CD8+ cell density in tissue sample at biopsy) × 100. One patient (1CB005) was excluded from the primary analysis because a tumor sample could not be collected within the allowable time frame before surgery after discontinuation

Fourteen of 15 participants had tissue samples available for primary endpoint analysis. CD8+ cell density was increased in tumor tissue from 12 participants (Figs. 2, 3). One participant (1CB005) was excluded from primary and secondary analyses because a tumor tissue sample could not be collected within the protocol-defined allowable time frame after study discontinuation. The median (range) percent change from baseline in CD8+ lymphocyte density following S-588410 administration was 149.37% (95% confidence interval, − 75.0%, 615.9%).

**Fig. 4:**
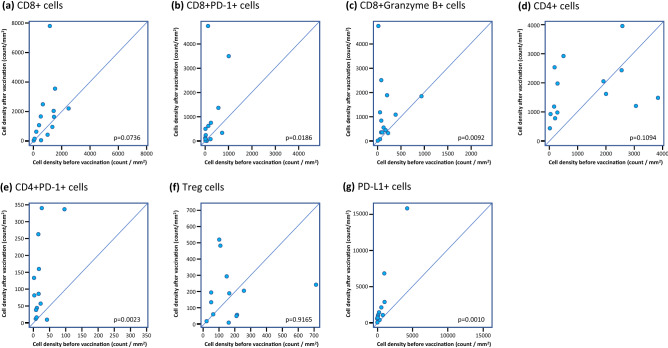
Plots of densities of cell subsets in tissue biopsy pre-vaccination versus those in the tumor tissue of surgical sections from patients vaccinated with S-588410. **a** CD8+ cells (*n* = 14), **b** CD8+PD-1+ cells (*n* = 14), **c** CD8+Granzyme B+ cells (*n* = 14), **d** CD4+ cells (*n* = 14), **e** CD4+PD-1+ cells (*n* = 14), **f** Treg cells (*n* = 13; number of specimens at post-vaccination in one patient [1CB023] was insufficient for staining for Treg cells), **g** PD-L1+ cells (*n* = 14). Differences were calculated for the full analysis set (FAS) and estimated with Wilcoxon signed-rank test. The FAS excluded one patient (1CB005) due to a deviation from the allowable time window defined in the clinical study protocol

The density of CD8+ cells in tumor tissue was not significantly increased (Fig. 4a), but the densities of both CD8+PD-1+ and CD8+Granzyme B+ cells increased significantly after vaccination (Fig. 4b, c). In addition, a significant increase of PD-1-expressing CD4+ cells was observed (Fig. 4d). However, changes in the density of whole CD4+ cells and regulatory T-cells (Treg cells) were variable between participants (Fig. 4e, f). Of note, PD-L1-expressing cell density increased after vaccination (Fig. 4g).

### Immune response analysis in blood

**Fig. 5:**
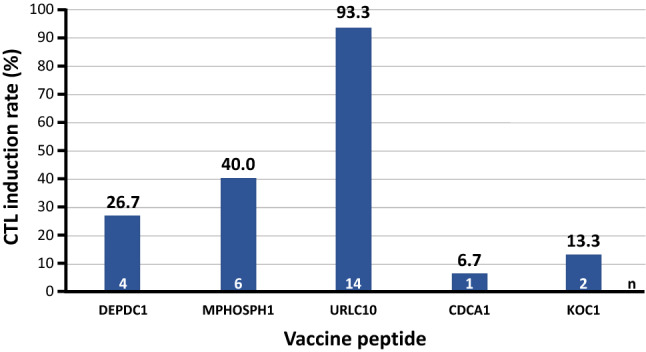
CTL induction in PBMCs post-vaccination with S-588410

CTL induction for at least one of the five constituent peptides was observed in all participants following S-588410 administration by ELISPOT assay after IVS PBMC culture (Fig. 5). CTL induction against at least three peptides was seen in two participants (Table 1). In the quantitative exploratory assessment by flow cytometry, URLC10 peptide-specific CD8+ T-cells in thawed PBMCs were increased in 11 out of 12 participants. The median (range) was 0.005% (0–0.02%) pre-vaccination and 0.06% (0.01–2.7%) post-vaccination. DEPDC1 peptide-specific CD8+ T-cells were also marginally increased in one participant (1CB023: pre-vaccination, 0%; post-vaccination, 0.21%).

**Fig. 6:**
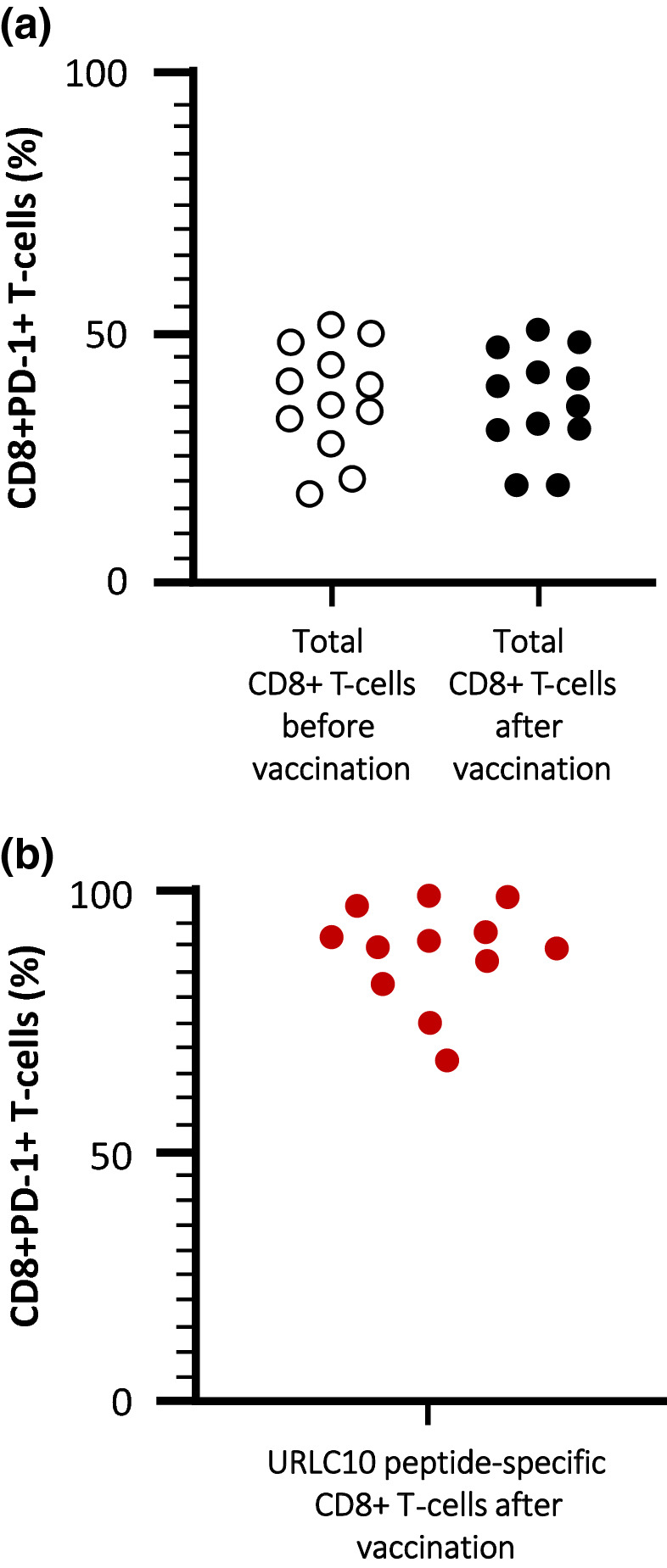
Immunological response measured in blood samples following S-588410 administration. **a** Proportion of total CD8+ T-cells expressing PD-1 before and after vaccination. **b** Proportion of URLC10 tetramer+CD8+ T-cells expressing PD-1 after vaccination

When examining the expression of functional/exhausted marker PD-1 by flow cytometry of URLC10 peptide-specific CD8+ T-cells and total CD8+ T-cells, URLC10 peptide-specific CD8+ T-cells induced higher expression of PD-1 compared with total CD8+ T-cells after vaccination (Fig. 6). Pre-vaccination PD-1 expression in URLC10 peptide-specific CD8+ T-cells was not determined because of the difficulty in harvesting a sufficient number of peptide-specific CD8+ T-cells to analyze.

### Detection of peptide-specific TCRs in tumor and blood

URLC10 peptide-specific TCRα and TCRβ sequences were examined in 10 participants with high URLC10 tetramer + CD8+ T-cell density after IVS culture. A sufficient number of sorted cells could not be obtained in two out of the 10 participants. Therefore, peptide-specific TCRα and TCRβ sequences were determined in eight participants for tracking (Supplemental Figure 2).

**Fig. 7:**
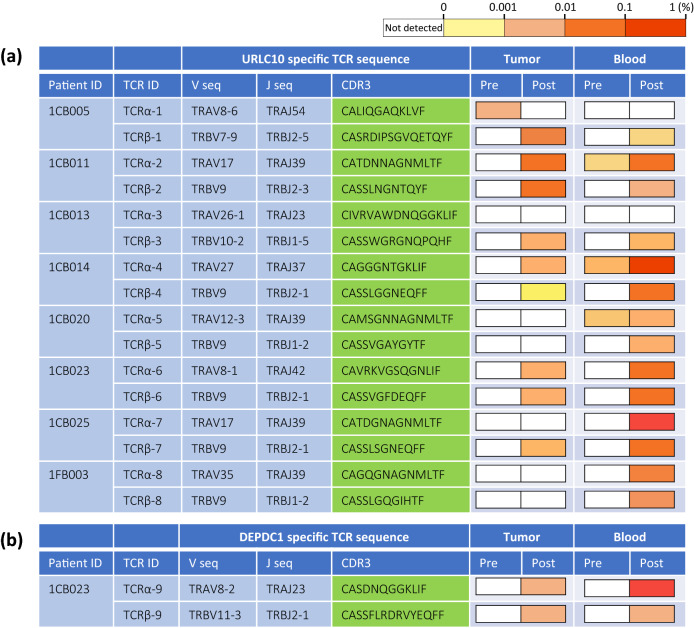
T-cell receptor (TCR) analysis. TCR sequences detected most frequently in the tetramer+CD8+ T-cell fraction after in vitro stimulation culture are exhibited. Heatmaps show the frequency of TCR sequence in tumor and blood pre- and post-vaccination. **a** URLC10 peptide-specific TCRα and TCRβ sequences were evaluated in eight patients.* **b** DEPDC1 peptide-specific TCRα and TCRβ sequences were evaluated in one patient. *1CB005 was accepted, even though the timing for obtaining a surgical sample deviated from the allowable time window defined in the clinical study protocol

The most frequent peptide-specific TCRα and TCRβ sequences were identified in blood samples post-vaccination, but were absent or detected less frequently in pre-vaccination samples. In tumor tissue, the peptide-specific TCRα and/or TCRβ sequences were observed post-vaccination, but not pre-vaccination, in six out of eight participants (except for a pre-vaccination peptide-specific TCRα in participant 1CB005) (Fig. 7a).

Similar results were observed using DEPDC1 peptide-specific TCRα and TCRβ sequences in the one DEPDC1 tetramer + participant (Fig. 7b). In addition, CTL clones that express TCRα and TCRβ, which were most frequently identified from the tetramer + CD8+ T-cell fraction, showed CTL activity for URLC10 or DEPDC1 peptide (Supplemental Figure 3).

## Discussion

In this exploratory study, we demonstrate that the five-peptide CPV S-588410 induces an immune response in tumor tissue from participants with esophageal cancer. Functional T-lymphocytes were increased, coupled with higher expression of PD-L1 in the TME after vaccination. Peptide-specific CTLs for all peptides were induced after a median five injections of S-588410, with at least one peptide being observed in all participants. URLC10-specific CTL was also detected in all participants and had the highest induction rate in this study, although CTL induction rates for other peptides were low. The lower rate of induction in this study may be explained by relatively low number of CPV injections compared with a previous study that demonstrated greater induction with a higher number of injections [13].

URLC10 CTL induction was highly prevalent (93.3%) and strong (grade +++) after S-588410 was administered a median 5 (range 3–14) times, in contrast to previous reports of low URLC10 CTL induction, requiring at least eight vaccine doses, in patients with advanced head and neck squamous cell carcinoma [14], advanced/recurrent non-small cell lung cancer [16] and advanced gastric cancer [17]. This suggests that patients with early-stage disease may respond to URLC10 CTL induction to a greater extent than patients with advanced disease, but DEPDC1- and MPHOSPH1-specific CTL induction was 60% in this study, which was similar to that seen after four vaccination doses in a previous study in patients with advanced bladder cancer [13]. It is unclear whether any variation in CTL induction relates to differences in immune escape mechanisms or response to tumor antigens.

IHC analysis showed that CD8 and CD4 cell functionality was significantly increased (based on results of CD8+ PD-1+ cells, CD8+ Granzyme B+ cells and CD4+ PD-1+ cells) despite a lack of a significant overall increase in CD8+ and CD4+ cell density. In addition, we found that PD-L1+ cells were significantly increased in participants with esophageal cancer during vaccination (Fig. 4g), while one-sided distribution was not shown in Treg (CD8-Foxp3+ CD25+) cells (Fig. 4f).

Assuming CD8+ T-cells infiltrate tumor tissue, it can be hypothesized that PD-L1 expression may be increased by IFN-γ produced by TILs induced by S-588410 administration. Our analysis found the same sequences of TCR-recognizing vaccine peptides in CD8+ cells from both blood and tumor samples and established CTL clones produced IFN-γ, providing further support for this hypothesis. Accumulation of CD4+ cells and CD8+ cells in the TME, and production of IFN-γ and Granzyme B, may also contribute to promoting an immune-inflamed TME [18].

S-588410 administration could also contribute to changes in the TME from an immune desert to an inflamed tumor. Accordingly, transformation of cancer-immune phenotypes by S-588410 may have therapeutic potential when combined with anti-PD-(L)1 antibodies in patients [19, 20]. When we evaluated PD-L1 expression in tumor cells by IHC, ≥ 5% of tumor cells were PD-L1+ in ten out of 15 participants after vaccination (data not shown). Fujimoto et al. [21] previously described the equivalence of the PD-L1 antibodies 22C3 (indicated in combination with pembrolizumab) and SP263 (used in our study). If the efficacy of pembrolizumab is confirmed in patients with ≥ 5% PD-L1+ tumor cells characterized by 22C3 in KEYNOTE-181 [22], PD-L1 induction by S-588410 may support combination therapy with a PD-(L)1 antibody.

S-588410 also induced PD-1-expressing CD4+ cells in the TME. It is generally accepted that 9–10-mer short peptides bind to major histocompatibility complex class I molecules and stimulate CD8+ cells through HLA class I [18]. Of note, it has been reported previously that survivin-derived short peptides induce CD4+ T-cell response and bind directly to HLA class II [23]. S-588410 may have indirect effects on CD4+ cells, but there are currently no data to show S-588410 has the ability to directly bind to HLA class II.

The role of CD4+ T-cells in the mechanism of immune response by short peptide vaccine remains controversial. Since the ratio of the PD-1 positivity among CD4+ cells is lower than that in CD8+ cells after vaccination, activation of CD4+ T-cells might be increased in the inflamed TME, elicited by functional CD8+ T-cells. The accumulation of effective CD4+ T-cells, rather than Treg cells, would therefore be important in enhancing the cytotoxic activity by CD8+ T-cells. Since PD-1+ CD4+ cells are thought to predict response to immune checkpoint inhibitors, the role of PD-1-expressing CD4+ T-cells should be explored in the future [20].

Establishing antigen expression is necessary for initiating any antigen-specific immune therapy. We detected the expression of all five CPV antigens in post-vaccination tumor tissue, with the exception of one participant without URLC10 expression and one participant with an unevaluable sample for DEPDC1 staining. Previously published data suggest that the level of antigen protein in post-vaccination tumors was lower than pre-vaccination tumors [10]. The number of injections in this earlier report was similar to the number in this study, suggesting the possibility that URLC10 expression was below the lower limit of detection after vaccination in this participant. HLA class I was also expressed in all participants. These results suggest the potential for the presentation of all five peptides from five antigen proteins in the TME.

The safety profile of S-588410 was acceptable. Injection site reaction was the most frequent AE, and its frequency and grade was similar to previous reports [7, 8, 10–14]. There were no serious treatment-related AEs; however, one participant withdrew from the study following an injection site reaction after three injections. This participant was included in the TCR repertoire analysis, despite tumor tissue being collected 26 days after withdrawal from the study, beyond the protocol-defined 14 days post-last injection. This decision was made based on notable CTL URLC10 induction, given only three injections in this participant, showing the highest population of URLC10-specific CD8+ cells in blood both before and after vaccination (URLC10 tetramer+: 0.02% → 2.7%). URLC10 TCRα sequences were detected in this participant’s tumor pre-vaccination, suggesting that URLC10 epitope-specific CD8+ T-cells might have a role of antitumor immunity without vaccination.

There are several limitations to this exploratory study. Firstly, study drug administration was planned based on the timing of surgery, resulting in differences in the number of injections. Secondly, an anti-CD3 antibody was not used for IHC, meaning there remains the possibility that CD8+ cells were not identified on T-lymphocytes. Furthermore, cells in a tumor with a peptide-specific TCR are not necessarily CD8+ cells. The small participant number and evaluable sample size should also be considered when interpreting results. With regard to the TCR sequence analysis, there was a bias toward cell proliferation in the fraction of tetramer + CD8+ cells.

In conclusion, vaccination with the CPV S-588410 induces functional CD8+ and CD4+ TILs and PD-L1 expression in esophageal cancer. The CD8+ TILs are derived from antigen-specific CTLs in blood, and an increase of CD8+ Granzyme B+ cells in tumors could lead to antitumor activity. The production of IFN-γ from CTLs may offer a mechanism for establishing an inflamed TME characterized by high PD-L1 expression. Therefore, a combination of S-588410 with anti-PD-(L)1 antibodies may be expected to have a synergistic effect.

### Electronic supplementary material

Below is the link to the electronic supplementary material.

Supplementary material 1 (PDF 514 kb)
