# Supplementary material for: Exploratory open-label clinical study to determine the S-588410 cancer peptide vaccine-induced tumor-infiltrating lymphocytes and changes in the tumor microenvironment in esophageal cancer patients
Source: Cancer Immunol Immunother. 2020 Jun 4;69(11):2247–57. doi: 10.1007/s00262-020-02619-3 (PMC7568713; doi:10.1007/s00262-020-02619-3)

Exploratory, open-label clinical study to determine the S-588410 cancer peptide vaccine-induced tumor-infiltrating lymphocytes and changes in the tumor microenvironment in esophageal cancer patients

Supplementary materials

Supplementary Methods 2
Supplemental Methods 1 2
Supplemental Methods 2 4
Supplemental Methods 3 6
Supplemental Methods 4 7
Supplementary Figures 10
Supplement Figure 1 10
Supplement Figure 2 11
Supplement Figure 3 12

Supplementary Methods

Supplemental Methods 1

List of antibodies for immunohistochemistry (IHC)

Target	Clone number	Chromogen	Supplier
CD8	4B11	Red	Leica Biosystems
CD4	4B12s	Brown	Leica Biosystems
CD25	4C9	Brown	Leica Biosystems
PD-1	NAT105	Blue	Abcam
PD-L1	SP263	Brown	Ventana
Foxp3	236A/E7	Green	Abcam
CTLA-4	F-8	Brown	Santa Cruz
Granzyme B	11F1	Blue	Leica Biosystems

Readouts from Panels after IHC

Fig. 4	Antibodies (Detection pattern)	Readout	Panel No.
A	CD8(positive), PD-1(any), CTLA-4(any)	CD8+	1
B	CD8(positive), PD-1(positive), CTLA-4(any)	CD8+PD-1+	1
C	CD8(positive), Granzyme B (positive), PD-L1(any)	CD8+Granzyme B+	2
D	CD8(negative), CD4(positive), PD-1(any)	CD4+	4
E	CD8(negative), CD4(positive), PD-1(positive)	CD4+PD-1+	4

F	CD8(negative), Foxp3(positive), CD25(positive)	Treg	3
G	CD8(any), Granzyme B(any), PD-L1(positive)	PD-L1+	2

Supplemental Methods 2

List of antibodies for immunohistochemistry (IHC)

Target	Name	Clone number	Supplier/manufacturer
DEPDC1	Anti-DEPDC1 antibody	16E9	Immuno-Biological Lab.
MPHOSPH1	Anti- MPHOSPH1 antibody	4-9A-5H	Immuno-Biological Lab.
URLC10	Ly-6K antibody	G-11	Santa Cruz Biotec.
CDCA1	Anti-Nuf2	4H9	MBL
KOC1	Monoclonal mouse anti-human IMP3	69.1	Agilent Technologies
HLA class I	Anti-HLA Class I ABC antibody	EMR8-5	Abcam

Scoring after IHC

Target cell	Tumor cells	
Allred score[1]	Proportion Score (PS)	0: 0%, 1: less than 1%. 2: 1% to 10%, 3: 10% to 1/3, 4: 1/3 to 2/3, 5: more than 2/3
	Intensity Score (IS)	0: Negative, 1: Weak, 2: Moderate, 3: Strong; The highest score was adapted.
	Total Score (TS) = PS + IS (range: 0–8)	
determination	Positive: total score 2-8, Negative: total score 0	

Reference

1. Allred DC, Harvey JM, Berardo M, Clark GM (1998) Prognostic and predictive factors in breast cancer by immunohistochemical analysis. Mod Pathol 11:155–168.

Supplemental Methods 3

CD8+ T-cell function assessment preparation

CD8+ T-cells were strictly detected in a gated cell population with cell surface markers (CD3+, CD8+, CD14-, CD19- and CD54-) purchased from BD Biosciences (San Jose, CA, USA) for CD3 (APC-H7, clone# SK7), CD8 (BV421, clone# SK1), CD19 (BV510, clone# SJ25C1), CD14 (BV510, clone# MψP9), CD56 (BV510, clone# NCAM16.2). In addition, peripheral blood mononuclear cells (PBMCs) were co-stained with allophycocyanin-conjugated tetramer for URLC10 (MBL, Nagoya, Aichi, Japan), phycoerythrin-conjugated tetramer for DEPDC1 (MBL, Nagoya, Aichi, Japan) and BV650-conjugated anti-PD1 antibody (clone# EH12.2H7; BioLegend, San Diego, CA, USA) for detecting peptide-specific functional CD8+ T cells.

Supplemental Methods 4

Acquirement of fraction of Tetramer+CD8+ T cells

After *in vitro* stimulation (IVS) culture, the cells were incubated with phycoerythrin-conjugated tetramer (Medical and Biological Laboratories, Nagoya, Aichi, Japan) on ice for 30 min. Following washing in Dulbecco's phosphate-buffered saline (DPBS; GIBCO, Waltham, MA, USA) with 0.5% bovine serum albumin (BSA; Iwai Chemicals, Tokyo, Japan), the cells were stained with a fluorescein isothiocyanate-conjugated anti-human CD8 antibody (clone RPA-T8; BD Biosciences, San Jose, CA, USA), allophycocyanin-conjugated anti-human CD3 antibody (clone UCHT1; BD Biosciences, San Jose, CA, USA) and phycoerythrin-Cy7-conjugated anti-human CD4 antibody (clone RPA-T4; BD Biosciences, San Jose, CA, USA) on ice for 20 min. The cells were washed in DPBS with 0.5% BSA and then stained with DPBS containing 0.1 µg/mL 4',6-diamidino-2-phenylindole (DAPI; BD Biosciences, San Jose, CA, USA). The cells were gated sequentially: lymphocytes (forward and side scatter), single cells, DAPI-/CD3+/CD4- cells, and then tetramer+CD8+ T-cells were sorted using SH800 cell sorter (Sony Biotechnology, San Jose, CA, USA).

Establishment of peptide-specific cytotoxic T-lymphocyte (CTL) clones and CTL assay

CTL clone was isolated from IVS culturing peripheral blood mononuclear cells (PBMCs) at post-vaccination by limiting dilution and expansion. Peptide-specific CTL clone was screened using interferon-γ (IFN-γ) enzyme-linked immunospot and expanded further using a rapid expansion method[1] with some modifications. Briefly, the cells were cultured in T25 flasks containing AIM-V (GIBCO, Waltham, MA, USA) supplemented with 5% human AB serum (MP Biomedicals, Tokyo, Japan), 40 ng/mL of anti-CD3 antibody (clone UCHT1; BD Biosciences, San Jose, CA, USA), 144IU/mL of interleukin-2 (IL-2; Novartis, Basel, Switzerland) and feeder cells. EB-3 and Jiyoye were used as feeder cells (5 x 10^6 cells each) after treating with mitomycin C (Kyowa Hakko Kirin, Tokyo, Japan). The half volume of culture medium was exchanged with fresh AIM-V supplemented with 5% human AB serum containing 72 IU/mL of interleukin-2 every 3 or 4 days. EB-3 (HLA-A3/Aw32, B-lymphoblastoid cell line) and Jiyoye (HLA-A32, B-lymphoblastoid cell line) were purchased from American Type Culture Collection (Manassas, VA, USA). To confirm the response of CTL clones to

URLC10 or DEPDC1 peptide, CTL clones (Responder) were co-cultured overnight with TISI cells (Stimulator) pulsed with or without peptide at the indicated ratio of Responder to Stimulator (R/S ratio). IFN-γ secretion was measured by enzyme-linked immunosorbent assay in triplicate. TISI (HLA-A*24:02, B-lymphoblastoid cell line) was purchased from the International Histocompatibility Working Group.

TCR repertoire analysis

Total RNA was extracted from tumor tissue and PBMC pre- and post-vaccination, as well as the sorted fraction of tetramer+CD8+ T cells after IVS culture using RNeasy mini kit or RNeasy micro kit (QIAGEN, Hilden, Germany). cDNAs with common 5'-RACE adapter were synthesized using SMARTScribe Reverse Transcriptase (Takara Bio USA, Inc. Kusatsu, Shiga, Japan). The cDNA libraries of TRAV and TRBV were acquired as previously described.[2] After adding the Illumina index sequences with barcode using the Nextera XT index kit v2 (Illumina, San Diego, CA, USA), the PCR products were sequenced using 300-bp paired-end reads on a MiSeq (Illumina, San Diego, CA, USA). TRAV and TRBV sequencing reads were mapped to the reference sequences in IMGT/GENE-DB for identifying V-(D)-J segments including complementary determining region 3 (CDR3).[3] We identified peptide-specific T-cell receptor (TCR) sequences in the fraction of tetramer+CD8+ T-cells and tracked their frequency in tumor tissue and PBMCs pre- and post-vaccination. TCR tracking was performed using software (ImmunoGrapher) developed by Cancer Precision Medicine, Inc (Kawasaki, Kanagawa, Japan).

Reference

1. Yoshimura S, Tsunoda T, Osawa R, Harada M, Watanabe T, Hikichi T, Katsuda M, Miyazawa M, Tani M, Iwahashi M, Takeda K, Katagiri T, Nakamura Y, Yamaue H (2014) Identification of an HLA-A2-restricted epitope peptide derived from hypoxia-inducible protein 2 (HIG2). PLoS One 9:e85267. https://doi.org/10.1371/journal.pone.0085267

2. Choudhury NJ, Kiyotani K, Yap KL, Campanile A, Antic T, Yew PY, Steinberg G, Park JH, Nakamura Y, O'Donnell PH (2016) Low T-cell receptor diversity, high somatic mutation

burden, and high neoantigen load as predictors of clinical outcome in muscle-invasive bladder cancer. Eur Urol Focus 2:445–452. https://doi.org/10.1016/j.euf.2015.09.007

3. Giudicelli V, Chaume D, Lefranc MP (2005) IMGT/GENE-DB: a comprehensive database for human and mouse immunoglobulin and T cell receptor genes. Nucleic Acids Res 33:D256–261. https:///doi.org/10.1093/nar/gki010

Supplementary Figures

Supplement Figure 1

T-cell receptor (TCR) analysis schema: Peptide-specific TCR sequences were acquired from tetramer+CD8+ T-cells using *in vitro* stimulation culturing of peripheral blood mononuclear cells (PBMCs) post-vaccination, followed by TCR sequencing. Frequency of the most dominant peptide-specific TCRα and TCRβ sequences in tetramer+CD8+ T-cells were tracked and TCR repertoire data was obtained from tumor and blood samples pre- and post-vaccination.

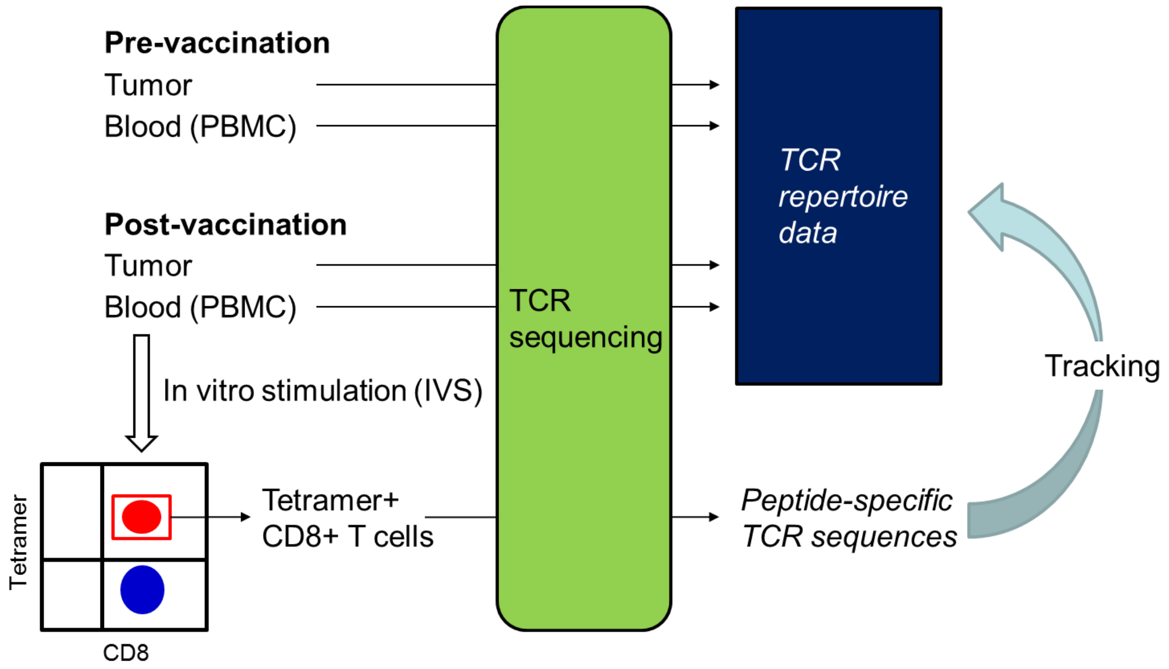

Supplement Figure 2

Pie charts show the frequency of T-cell receptor (TCR) sequences in the tetramer+CD8+ T cell fraction after *in vitro* stimulation culture. Gray zone indicates sum of TCR sequences of <1% frequency. Most frequent TCR sequences are shown by TCR ID (TCRα-x or TCRβ-x). Asterisks (*) are TCR sequences identified from peptide-specific cytotoxic T-lymphocyte clones. (A) URLC10 tetramer+CD8+ T cells fraction were examined in eight participants. (B) DEPDC1 tetramer+CD8+ T cell fraction was examined in one participant.

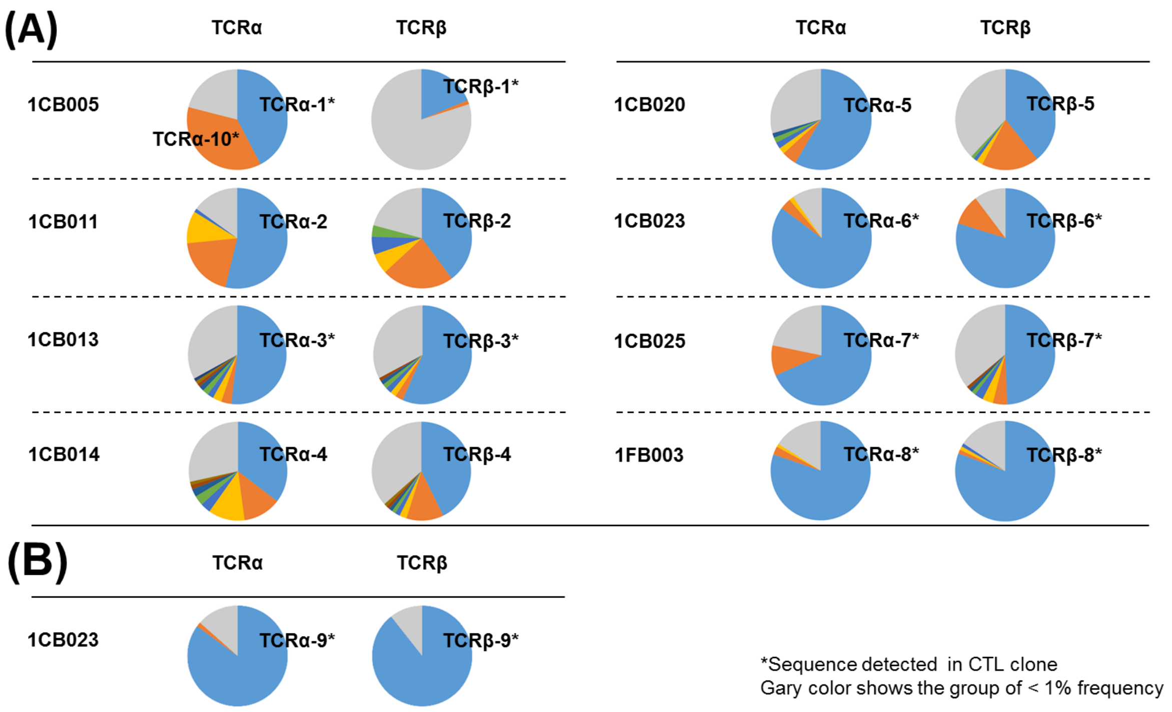

Supplement Figure 3

Cytotoxic T-lymphocyte (CTL) clones showed specific responses for URLC10 or DEPDC1 peptide. T-cell receptors (TCRs) were identified from these CTL clones. (A) URLC10 peptide-specific CTL clones were established from five patients. Two TCRαs were identified in 1CB005. They were two most frequently detected sequences from the tetramer+CD8+ T cell fraction after *in vitro* stimulation culture. (B) A DEPDC1 peptide-specific CTL clone was established from one patient.

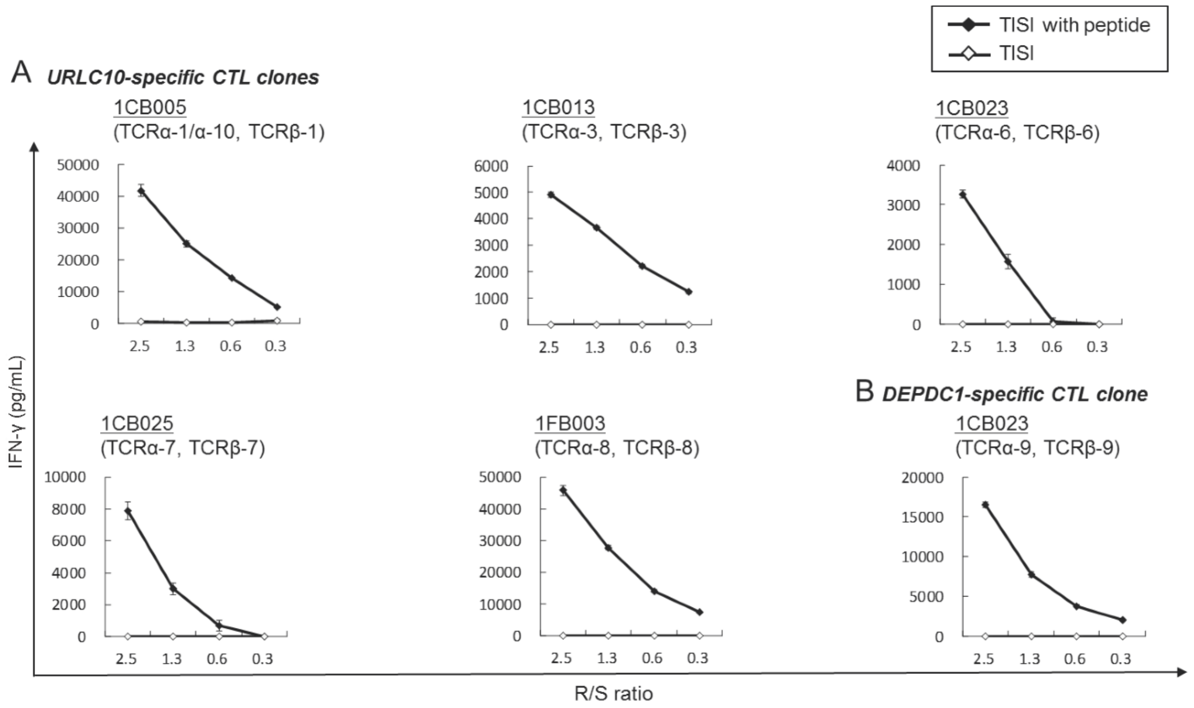

Supplement: Supplementary file 1 — Supplementary material 1 (PDF 514 kb) [file 262_2020_2619_MOESM1_ESM.pdf]
